# Novel nanocomposites of Ni-Pc/polyaniline for the corrosion safety of the aluminum current collector in the Li-ion battery electrolyte

**DOI:** 10.1038/s41598-021-91688-0

**Published:** 2021-06-11

**Authors:** M. A. Deyab, G. Mele, E. Bloise, Q. Mohsen

**Affiliations:** 1 Egyptian Petroleum Research Institute (EPRI), Nasr City, Cairo, Egypt; 2 Department of Engineering for Innovation, University of Salento, via Monteroni, 73100 Lecce, Italy; 3 Department of Chemistry, College of Sciences, Taif University, Taif, Saudi Arabia

**Keywords:** Chemistry, Electrochemistry

## Abstract

In electrochemical energy storage systems, Li-ion batteries have drawn considerable interest. However, the corrosion of the aluminum current collector in the LiN(SO_2_CF_3_)_2_ electrolyte has a major effect on battery efficiency. To protect the current collector from the corrosive action of the LiN(SO_2_CF_3_)_2_ electrolyte, new nanocomposites based on Ni(II)tetrakis[4-(2,4-bis-(1,1-dimethyl-propyl)-phenoxy)]phthalocyanine (Ni-Pc) and polyaniline matrix (PANI) (i.e. PANI@Ni-Pc composites) are coated on the aluminum current. SEM, XRD, and EDS were used to characterize the PANI@Ni-Pc composite. This method represents a novel approach to the production of Li-ion batteries. Electrochemical tests show that the PANI@Ni-Pc composites can protect aluminum from corrosion in LiN(SO_2_CF_3_)_2_. The output of PANI@Ni-Pc composites is influenced by the Ni-Pc concentration. The composite PANI@Ni-Pc is a promising way forward to build high-stability Li-Ion batteries.

## Introduction

Li-ion battery (LIB) can be categorized as rechargeable battery^1,2^. LIBS are mainly used for electric vehicles applications. Among the main component in the LIBs is aluminum, which used as a current collector for cathode electrodes in LIBs^3,4^. The main problem facing aluminum current collector is corrosion due to contact with LiN(SO_2_CF_3_)_2_^5–7^. Indeed, the low of F-containing film formed on the current collector causes the low corrosion resistance of Al surface^8^. In addition, the N(SO_2_CF_3_)_2_ anion increases the solubility of passive layer (Al_2_O_3_), leading to uncover Al surface^9^. This causes the direct contact between current collector and corrosive electrolyte^10,11^ and leads to the loss in LIBs performance. Previous works have been conducted to overcome the corrosion of Al surface in the electrolytes of LIB by various strategies. The first strategy is using suitable additives to electrolyte to inhibit the Al corrosion. Louis et al.^12^ used fumed silica as electrolyte additive to suppress the Al corrosion in LIB. They indicated that silica particles form gel layer on the Al surface and this prevents the corrosive action of electrolyte. In the recent work by Zhuang et al.^13^ we found that the water impurity in the battery electrolyte was removal by adding trimethylsilyl (trimethylsiloxy) acetate*.* This leads to suppress the corrosion of battery electrodes and current collector.

The second strategy depends on the replacing the high corrosive electrolytes by less corrosive electrolytes such as organic compounds or ionic liquids. Based on the Theivaprakasam work^14^, the using of hybrid electrolytes composed of an ionic liquid and a conventional electrolyte mixture have the great effect on the passive layer on the Al surface and leads to the decrease in the Al corrosion. Richard Prabakar et al.^15^ used another strategy to protect the Al current collector from corrosion based on the formation of graphene oxide (GO) on the Al surface. In this work GO can remain the Al_2_O_3_ passive layer on the Al surface for long time without degradation.

Here, we developed new and unique strategy to protect the Al current collector from the corrosive action of LiN(SO_2_CF_3_)_2_ electrolyte. This strategy depend on the coating the Al surface by new nanocomposite based on Ni(II)tetrakis[4-(2,4-bis-(1,1-dimethyl-propyl)-phenoxy)]phthalocyanine (Ni-Pc) and polyaniline matrix (PANI).

PANI was developed in recent years to protect the metal against corrosion with reasonable anti-corrosion performance and high conductivity^16–18^. Because the PANI coating is eco friendly and simple to prepare, it has gained widespread attention around the world^19^. Despite the long work on the anti-corroding properties of PANI, many problems for PANI-based coatings still remain to be solved, such as their porous structure and poor barrier effect^20,21^. Designing and developing PANI-based composite systems, which typically consist of a PANI and various nano particle fillers such as Ni-Pc, is one effective strategy for overcoming the aforementioned disadvantages. This work would open new strategy for improving the performance of LIBs using nanocomposites coatings.

## Materials and methods

### Materials

LiN(SO_2_CF_3_)_2_ in ethylene carbonate EC/ dimethyl carbonate DMC 50/50 (v/v); battery grade was obtained from Merck KGa. Battery Grade Al foil (purity > 99%, 40 mm thick) was supplied from Aldrich Chemicals. The used chemicals such as N-Methyl-pyrrolidone (NMP) and polyaniline (emeraldine salt, PANI) were supplied from Sigma Aldrich Co.

### Preparation of PANI@Ni-Pc composites

Ni-Pc has been prepared as described from previous work^22^. We used direct solid-state mixing method to prepare PANI@Ni-Pc composites^23,24^. Here, 1.0 g of PANI was mechanically mixed with different percentages of Ni-Pc (i.e. 0.2%, 0.4% and 0.6% Ni-Pc). The compositions were ground into fine particles. PANI@Ni-Pc powered was dispersed in NMP by sonication for 3.0 h. Finally, we prepared three PANI@Ni-Pc composites (i.e. PANI@0.2Ni-Pc, PANI@0.4Ni-Pc, and PANI@0.6Ni-Pc). Higher percentages of Ni-Pc (i.e. > 0.6%) are limited by uniformity and nano-particles re-aggregation after mechanical removal by stirring. Spin Coaters (Specialty Coating Systems Inc.) was used to apply a uniform layer of PANI@Ni-Pc composites on the clean surface of Al foil. The coated Al foil samples were dried at 353 K for 6.0 h. The coating has a thickness of around 35 ± 5 µm. (measured by micrometer, B.C.Ames).

### Electrochemical measurements

Potentiostat/Galvanostat (Gamry 3000) connected with ZWIEW software was used for all electrochemical measurements. We used a 3-electrodes beaker cell as described in pervious works^25,26^, where Li foils act as counter and reference electrodes. The working electrodes are Al foils coated with pristine PANI and PANI@Ni-Pc composites. The conditions of cyclic voltammetry (CV) experiments are potential range 1–5 V (vs. Li/Li^+‏^), scan rate 10 mV s^−1^, temperature 303 K. The conditions of chronoamperometry (CA) experiments are: applying a potential step 4.0 V (vs. Li/Li^+‏^), time 4000 s, and temperature 303 K. The conditions of electrochemical impedance spectroscopy (EIS) experiments are open circuit potential; frequency ranges 10 kHz–1.0 Hz, applied amplitude 10 mV, and temperature 303 K. The EIS measurements started after the electrode polarized for 60 min.

The charge/discharge (C–D) test of LIB was conducted using Lithium Battery Materials Cell Kit (Gamry Instruments). The discharge capacity of the LIB was recoded in the potential range 2.5–4.0 V and at the rate of 1 C.

### Materials characterization

The Materials characterizations were investigation by using scanning electron microscopy (SEM) (model: JEOL-JSM-6510), X-ray spectroscopy (EDS) (JEOL-JED-2300T) and X-ray diffractometer (XRD) (Panalytical diffractometer X’pert MPD).

## Results and discussion

### PANI@Ni-Pc composite characterizations

**Figure 1:**
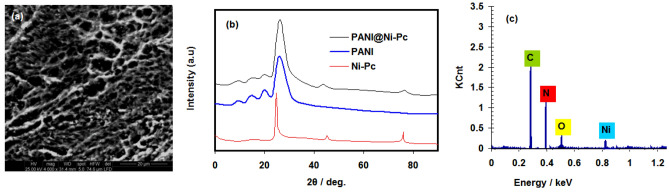
(**a**) SEM image of PANI@Ni-Pc composite, (**b**) XRD pattern of PANI, Ni-Pc and PANI@Ni-Pc composite, and (**c**) EDS pattern of PANI@Ni-Pc composite.

SEM image of PANI@Ni-Pc composite is presented in Fig. 1a. The surface PANI@Ni-Pc composite has homogeneous texture between tubular PANI nanostructures and Ni-Pc nanosheets.

XRD pattern of PANI, Ni-Pc and PANI@Ni-Pc composite is presented in Fig. 1b. The diffraction peaks observed at 2θ = 25.9, 20, 14.7, and 9.4, according with literature data^27^, indicates that bare PANI has semi-crystalline nature. Differently, the Ni-Pc showed neat characteristic peaks at 2θ = 24.8, 45.2, and 76.3 indicating a greater crystallinity degree that it loses when mechanically mixed with the PANI matrix to form the nanocomposite. Indeed, the XRD pattern of PANI@Ni-Pc composite reveals the presence peaks for both PANI and Ni-Pc but with a broader shape similar to unmodified PANI, which also refers a semi-crystalline nature for the composite. The shift from Ni-Pc could be ascribed to the loss of Ni-Pc when it is mixed with PANI.

EDS pattern (see Fig. 1c) confirms the chemical composition of PANI@Ni-Pc composite with the presence of C, N, O and Ni peaks.

### CV studies

**Figure 2:**
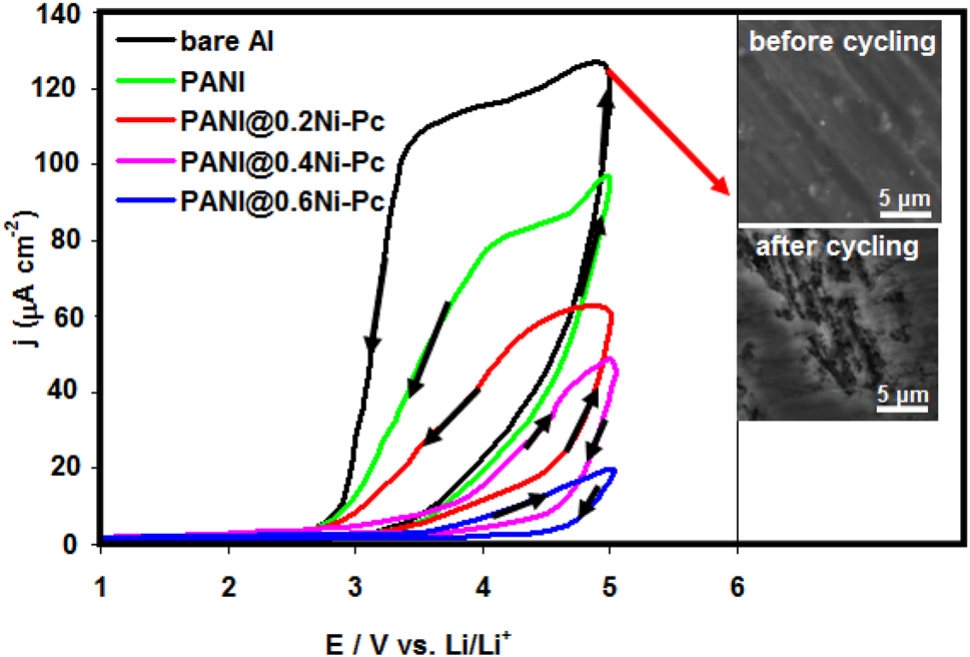
CV curves for pristine Al foil and coated Al foil with pristine PANI and PANI@Ni-Pc composites in LiN(SO_2_CF_3_)_2_/EC:DMC (1:1 by vol.). Inset image shows SEM image of pristine Al foil in LiN(SO_2_CF_3_)_2_ before and after cycling.

**Scheme 1:**
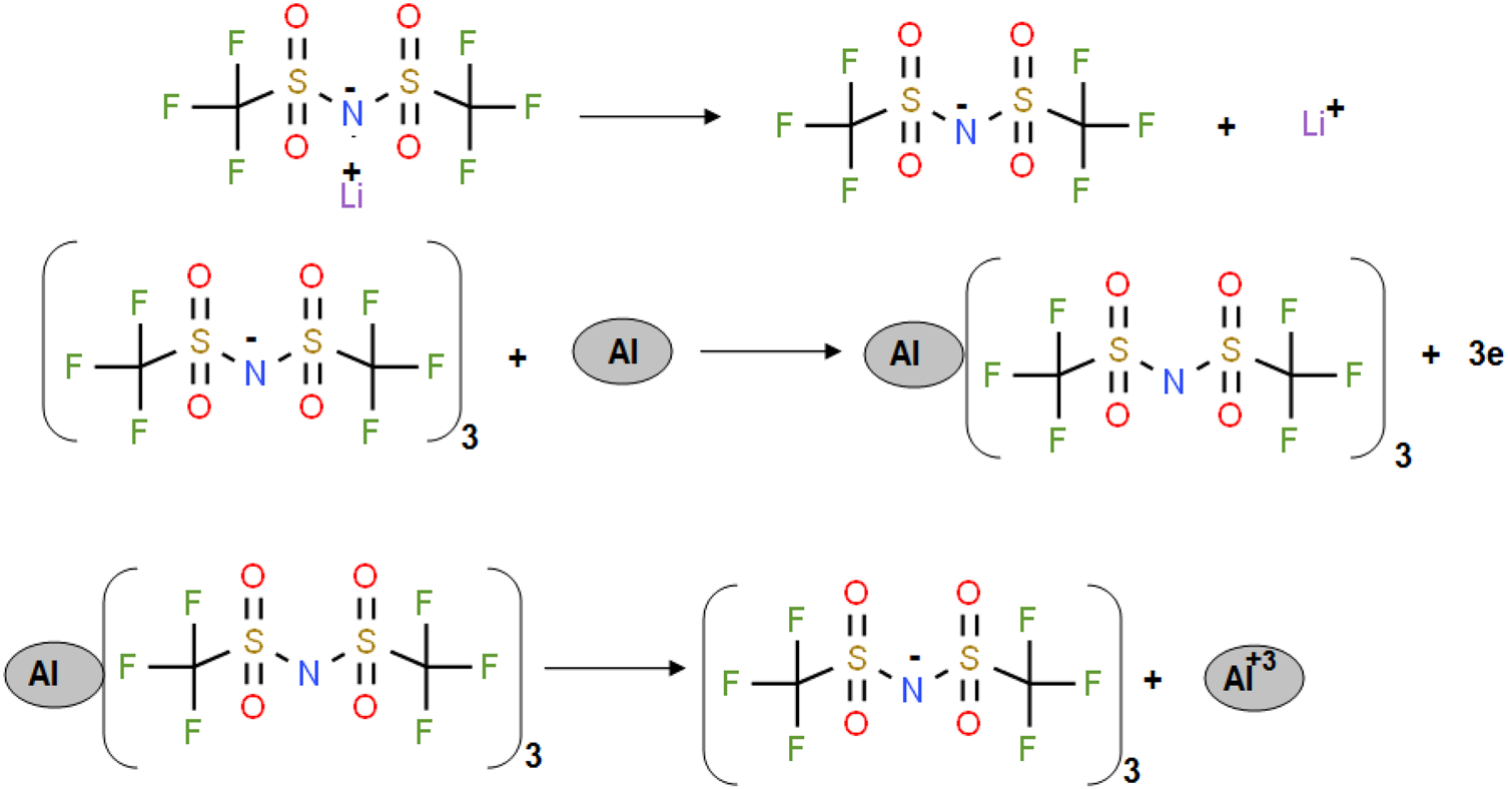
The corrosion of aluminum in 1.0 M LiN(SO_2_CF_3_)_2_ electrolyte.

Figure 2 shows CV behavior for pristine Al foil and coated Al foil with pristine PANI and PANI@Ni-Pc composites in 1.0 M LiN(SO_2_CF_3_)_2_/EC:DMC (1:1 by vol.). In the case of pristine Al foil, we noted a fast and sudden change in the passive region at 3.4 V. This change includes the steeply increase in the current density till 5.0 V. The huge anodic current at the potential region 3.4–5.0 V represents the pitting corrosion of Al foil and formation Al^3+^. Furthermore, the hysteresis loop was formed during the reverse scanning, indicating the occurring the pitting corrosion^28,29^ (see inset SEM images in Fig. 2). The corrosion steps can be represented in the scheme 1^12^:

As shown in the Fig. 2, the corrosion inhibition of coated Al foil with pristine PANI was achieved. The CV of the pristine PANI coated Al foil, on the other hand, shows an unstable pattern, suggesting that the permeated aggressive ions have started the corrosion process. The lower anodic current for coated Al foil with PANI@Ni-Pc composites comparing with pristine Al foil and pristine PANI confirmed the anticorrosion nature of the as-prepared composites^30^, suggesting that the PANI@Ni-Pc composites are able to protect Al current collector from corrosion in 1.0 M LiN(SO_2_CF_3_)_2_. The performance of PANI@Ni-Pc composites depends on Ni-Pc concentration. The CV reveals that the PANI@0.2Ni-Pc can decrease the anodic current but it can not overcome completely on the hysteresis loop (i.e. pitting corrosion). The hysteresis loop was disappeared in the case of PANI@0.4Ni-Pc and PANI@0.6Ni-Pc. Furthermore at the highest Ni-Pc percentage (i.e. PANI@0.6Ni-Pc), we got the lowest anodic current. This means that coated Al foil with PANI@0.6Ni-Pc composite gives the best anti-corrosion properties.

### CA studies

**Figure 3:**
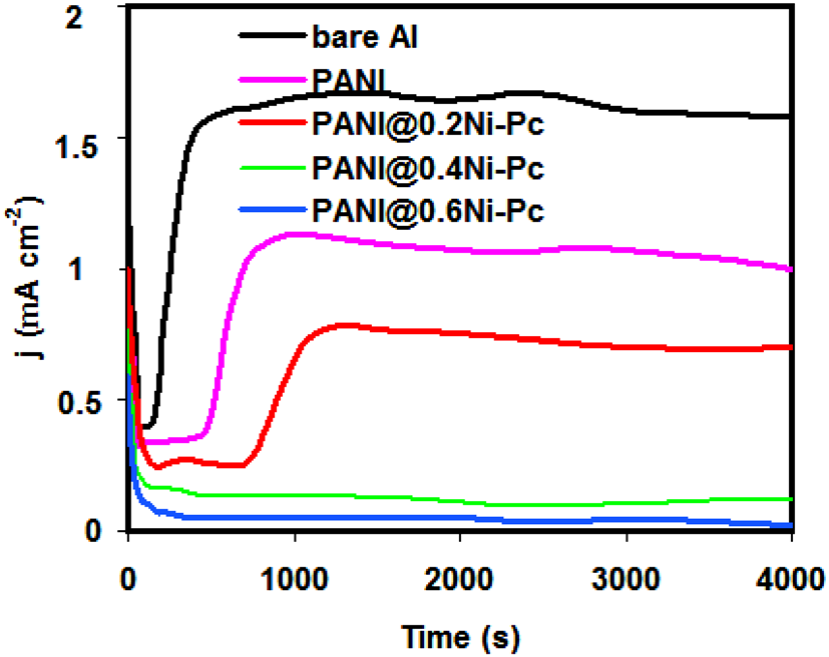
CA curves for pristine Al foil and coated Al foil with pristine PANI and PANI@Ni-Pc composites in LiN(SO_2_CF_3_)_2_/EC:DMC (1:1 by vol.).

Figure 3 shows CA behavior for pristine Al foil and coated Al foil with pristine PANI and PANI@Ni-Pc composites in 1.0 M LiN(SO_2_CF_3_)_2_/EC: DMC (1:1 by vol.). After applying 4.0 V, the current of pristine Al foil quickly increased to 1.6 mA cm^−2^ in short time (380 s) and progressively reach to equilibrium state within experiment time (i.e. 4000 s). In this case, the adsorbed N(SO_2_CF_3_)_2_^−^ ions can permeate inside Al oxide layer (i.e. passive layer) and reach the base Al foil surface and start pitting corrosion^31^. This behavior refers to the continuous of the corrosion process of Al foil in 1.0 M LiN(SO_2_CF_3_)_2_ after short time. In addition, the passive layer is not able to protect the Al foil from corrosion.

Compared with pristine Al foil and pristine PANI, the current density for coated Al foil with PANI@0.2Ni-Pc composites was dropped to less value. Besides, the time required to start pitting corrosion was longer (i.e. 750 s). In the case of PANI@0.4Ni-Pc and PANI@0.6 Ni-Pc, the steady-state of the current density reached 0.22 mA cm^−2^ and 0.13 mA cm^−2^, respectively, without pitting corrosion signs. This confirms that PANI@Ni-Pc composites are able to protect the Al foil from corrosion. Also the ability of PANI@Ni-Pc composites to minimize the anodic current is closely related to the concentration of Ni-Pc. Thus, the high of Ni-Pc concentration (i.e. 0.6%) is highly beneficial for inhibiting the Al foil corrosion. which is symmetrical with the results from CV studies.

### EIS studies

**Figure 4:**
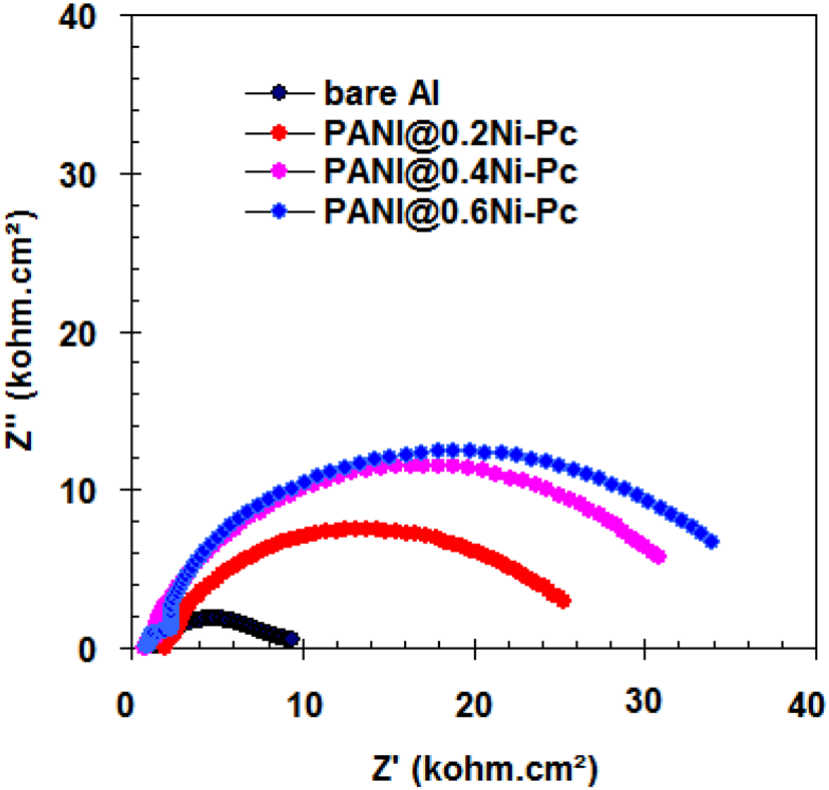
Nyquist plots for pristine Al foil and coated Al foil with PANI@Ni-Pc composites in LiN(SO_2_CF_3_)_2_/EC:DMC (1:1 by vol.).

**Figure 5:**
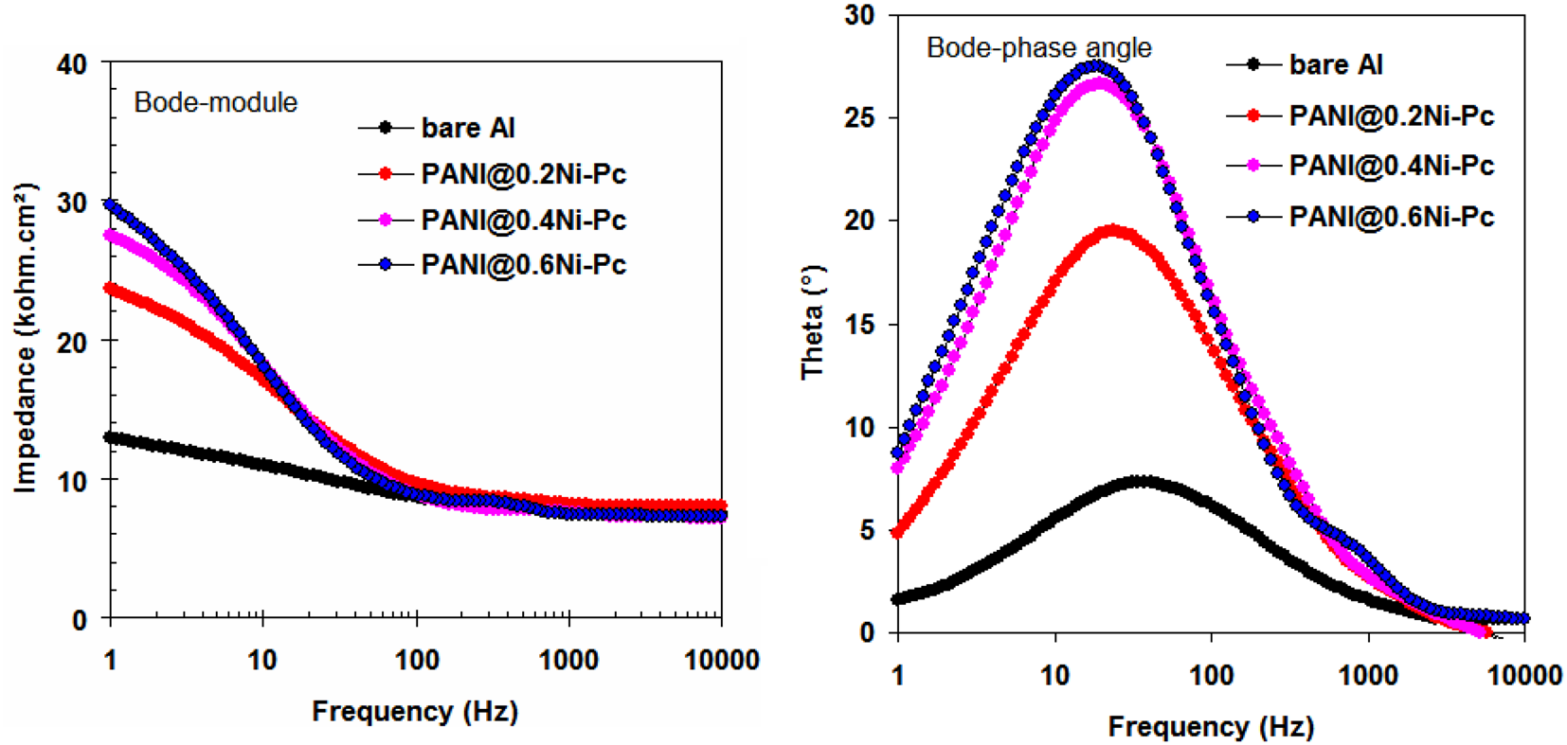
Bode-module and Bode-phase angle plots for pristine Al foil and coated Al foil with PANI@Ni-Pc composites in LiN(SO_2_CF_3_)_2_/EC:DMC (1:1 by vol.).

Figure 4 represents Nyquist behavior for pristine Al foil and coated Al foil with PANI@Ni-Pc composites in 1.0 M LiN(SO_2_CF_3_)_2_/EC: DMC (1:1 by vol.). Also the Bode-module and Bode-phase angle plots are presented in Fig. 5. In all cases (i.e. pristine and coated Al foils), the Nyquist spectra exhibit two semi-circles in the high and low frequency regions. The first semi-circles at high frequency region is related to the passive layer formation and/or composites coating layer film, and the second semi-circles at low frequency region is related to the charge transfer processes on both cathode and anode^32,33^.

**Figure 6:**
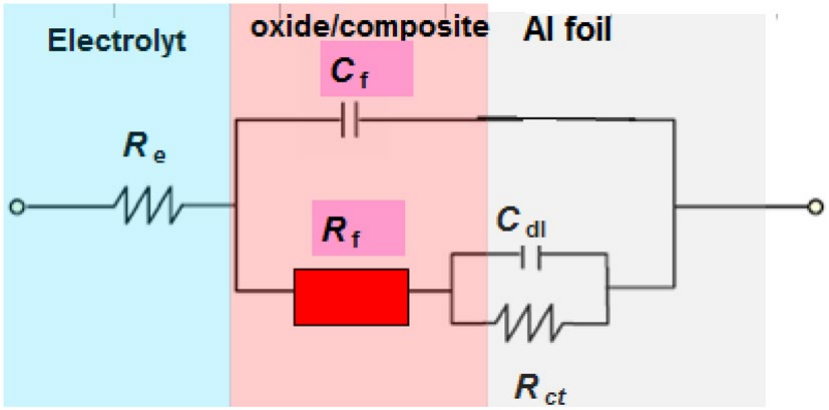
Equivalent circuit fitted for EIS spectra.

The fitted equivalent circuit for EIS spectra is shown in Fig. 6. In the circuits, *R*_e_ = electrolyte resistance, *R*_f_ = film resistance, *C*_f_ = film capacitance, *R*_ct_ = the charge transfer resistance and *C*_dl_ = double-layer capacitance. For the pristine Al foil, *R*_f_ = 2.6 kΩ cm^2^, *C*_f_ = 26.5 μF cm^−2^, *R*_ct_ = 6.3 kΩ cm^2^ and *C*_dl_ = 6.05 μF cm^−2^. We noted that the resistance values (*R*_f_ and *R*_ct_) obtained from coated Al foils increased comparing with pristine Al foil, indicating the excellent anti-corrosion capability of PANI@Ni-Pc composites. The highest resistance values (*R*_f_ = 5.4 kΩ cm^2^ and *R*_ct_ = 33.2 kΩ cm^2^) were recorded for the coated Al foils with PANI@0.6Ni-Pc composite, indicating the better anti-corrosion properties for PANI@0.6Ni-Pc composite.

The film capacitance is due to the passive film formation and /or composites film on Al foil^34,35^. Thus the *C*_f_ value (*C*_f_ = 26.5 μF) for the pristine Al foil decreases with using coated Al foil (*C*_f_ = 12.3 μF for PANI@0.2Ni-Pc, *C*_f_ = 5.4 μF for PANI@0.4Ni-Pc, *C*_f_ = 1.2 μF for PANI@0.6Ni-Pc). Generally, PANI@Ni-Pc composites enhance the physical barrier and coated film for Al foils.

### C–D studies

**Figure 7:**
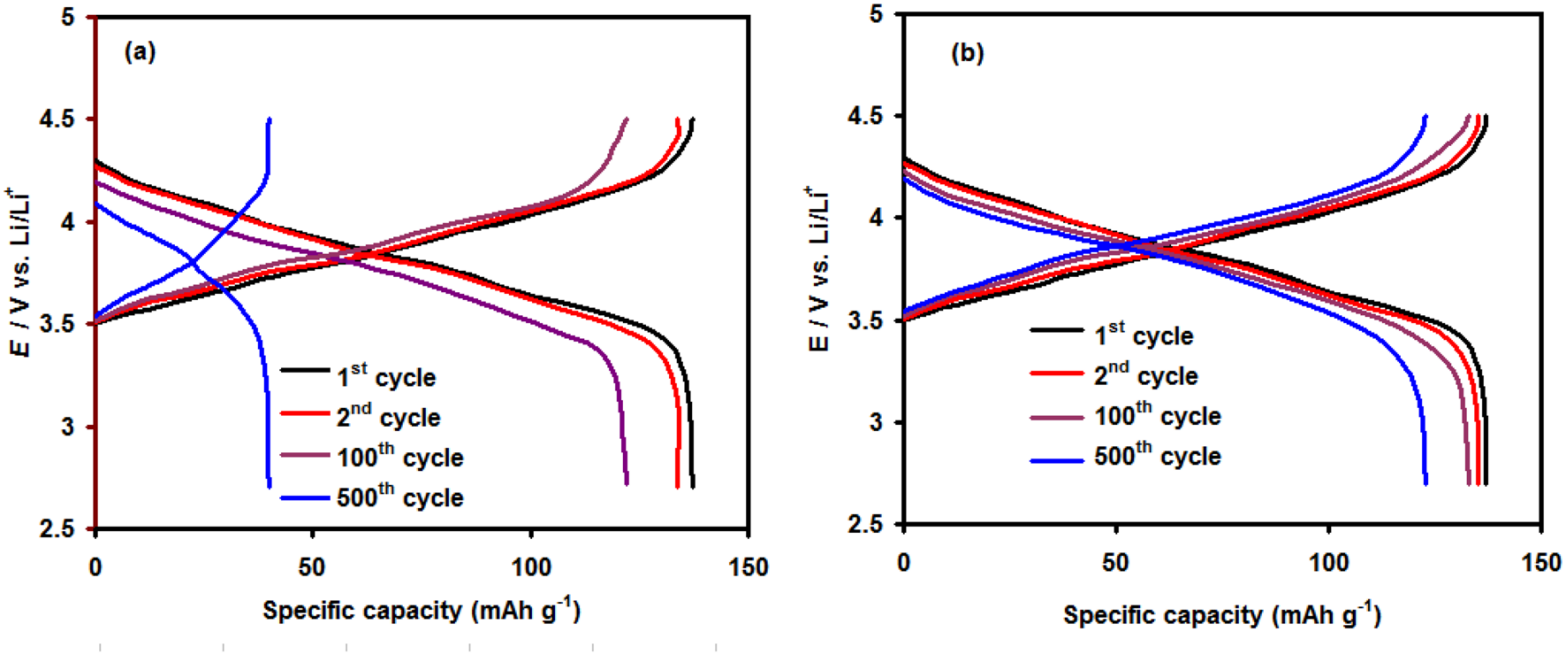
Charge and discharge curves of LIB (at 2C) with pristine Al foil (**a**) and coated Al foil (PANI@0.6Ni-Pc composite) (**b**) at various cycles.

To evaluate the influence of PANI@Ni-Pc composites on the overall performance of LIBs, C–D tests were conducted at a rate of 2C using pristine Al foil and coated Al foil. Figure 7 compares the LIB's C–D curves at various cycles. The results show that the LIB with coated Al foil by PANI@0.6Ni-Pc composite has a higher discharge capacity and better capacity retention than the pristine Al foil. The cell with the PANI@0.6Ni-Pc composite has a higher specific capacity (92% of initial capacity after 500 cycles) than cell with pristine Al foil. This means that the PANI@0.6Ni-Pc composite plays a vital role in protecting Al foil from corrosion in 1.0 M LiN(SO_2_CF_3_)_2_ electrolyte during cycling, leading to high stability and super performance of LIBs.

### Mechanism of action of PANI@Ni-Pc composites

Generally, Al foil corrosion in the LIBs leads to numerous troubles many problems such as passive layer formation, electrolyte contamination and fast capacity fading^36–38^. Here, the above results confirm that the using of coated Al foil with PANI@Ni-Pc composites can protect the Al foil from the corrosion in 1.0 M LiN(SO_2_CF_3_)_2_ electrolyte. PANI coating represents the first line of defense in the isolation of Al foil from the electrolyte, leading to the less contact points between Al foil and the surrounding^39^. Unfortunately, the use of PANI coating alone is not enough to protect the Al foil from corrosion. This is due to the porosity of PANI coating, which allows for the corrosive electrolyte transit^40^. The incorporation of nano-particles of Ni-Pc inside the PANI matrix has the great effect on the anti-corrosion properties of PANI@Ni-Pc composites and consequently on the LIBs stability. Nano-particles of Ni-Pc fulfill their objectives through the following functions:

i. Reducing the porosity of PANI matrix by filling the empty spaces inside the coating matrix^41^.
ii. Increasing the tortuosity inside PANI matrix, leading to the hinder the movement of corrosive ions inside the PANI matrix^42^.
iii. Reducing the deformation of the PANI matrix, leading to the strong adhesion strength and good protective layer^43^.
iv. The strong adhesion forcer between Al foil and PANI@Ni-Pc is due to the ionization potentials of central atoms (i.e. Ni = 8.32 eV)^44,45^.

The PANI@Ni-Pc composites keep the surface of the Al foil clean and free from the oxide and corrosion products and this helps the Al foil to work as current collector with high efficiency^46^. In addition the high conductivity of PANI helps to make the good electrical contact for current collector during battery discharge.

## Conclusions

In this work, we report PANI@Ni-Pc composites as new coatings for Al current collector in LIBs. Our investigations showed that PANI@Ni-Pc composites are able to protect the Al current collector from corrosion in 1.0 M LiN(SO_2_CF_3_)_2_ electrolyte. This was confirmed by CV, CA, and EIS measurements. The pitting corrosion on the Al foil surface was disappeared in the case of PANI@0.4Ni-Pc and PANI@0.6Ni-Pc. Furthermore, we got the highest resistance values (*R*_f_ = 5.4 kΩ cm^2^ and *R*_ct_ = 33.2 kΩ cm^2^) for coated Al foils with PANI@0.6Ni-Pc composite, indicating the better anti-corrosion properties for PANI@0.6Ni-Pc composite. Our work verified that PANI@Ni-Pc composites are effective as a new strategy for improving the LIBs performance.
